# Paternal chromosome elimination of inducer triggers induction of double haploids in *Brassica napus*

**DOI:** 10.3389/fpls.2023.1256338

**Published:** 2023-10-30

**Authors:** Shihui Zhao, Liangjun Huang, Qing Zhang, Ying Zhou, Meicui Yang, Haoran Shi, Yun Li, Jin Yang, Chao Li, Xianhong Ge, Wanzhuo Gong, Jisheng Wang, Qiong Zou, Lanrong Tao, Zeming Kang, Zhuang Li, Chaowen Xiao, Qiong Hu, Shaohong Fu

**Affiliations:** ^1^ Chengdu Academy of Agriculture and Forestry Sciences, Chengdu Research Branch, National Rapeseed Genetic Improvement Center, Chengdu, China; ^2^ Agricultural College, Sichuan Agricultural University, Chengdu, China; ^3^ College of Life Sciences, Sichuan University, Chengdu, China; ^4^ Oil Crops Research Institute, Chinese Academy of Agricultural Science, Wuhan, China; ^5^ College of Plant Science and Technology of Huazhong Agricultural University, Wuhan, China

**Keywords:** inducer line, chromosome elimination, embryo, paternal chromosome, *Brassica napus* L.

## Abstract

A synthetic octoploid rapeseed, Y3380, induces maternal doubled haploids when used as a pollen donor to pollinate plant. However, the mechanism underlying doubled haploid formation remains elusive. We speculated that double haploid induction occurs as the inducer line’s chromosomes pass to the maternal egg cell, and the zygote is formed through fertilization. In the process of zygotic mitosis, the paternal chromosome is specifically eliminated. Part of the paternal gene might have infiltrated the maternal genome through homologous exchange during the elimination process. Then, the zygote haploid genome doubles (early haploid doubling, EH phenomenon), and the doubled zygote continues to develop into a complete embryo, finally forming doubled haploid offspring. To test our hypothesis, in the current study, the octoploid Y3380 line was back bred with the 4122-*cp4-EPSPS* exogenous gene used as a marker into hexaploid Y3380-*cp4-EPSPS* as paternal material to pollinate three different maternal materials. The fertilization process of crossing between the inducer line and the maternal parent was observed 48 h after pollination, and the fertilization rate reached 97.92% and 98.72%. After 12 d of pollination, the presence of *cp4-EPSPS* in the embryo was detected by *in situ* PCR, and at 13–23 d after pollination, the probability of F_1_ embryos containing *cp4-EPSPS* gene was up to 97.27%, but then declined gradually to 0% at 23–33 d. At the same time, the expression of *cp4-EPSPS* was observed by immunofluorescence in the 3rd to 29th day embryo. As the embryos developed, *cp4-EPSPS* marker genes were constantly lost, accompanied by embryonic death. After 30 d, the presence of *cp4-EPSPS* was not detected in surviving embryos. Meanwhile, SNP detection of induced offspring confirmed the existence of double haploids, further indicating that the induction process was caused by the loss of specificity of the paternal chromosome. The tetraploid-induced offspring showed infiltration of the induced line gene loci, with heterozygosity and homozygosity. Results indicated that the induced line chromosomes were eliminated during embryonic development, and the maternal haploid chromosomes were synchronously doubled in the embryo. These findings support our hypothesis and lay a theoretical foundation for further localization or cloning of functional genes involved in double haploid induction in rapeseed.

## 1 Introduction

In maize (*Zea mays* L.), Coe (1959) discovered that Stock-6, when used as a pollen donor for F_1_ hybrid, produced 2.29% parthenogenetic haploids in its offspring, thus paving the way for induced in-planta haploids to be used in rapid crop breeding. Similarly, the crossing of *Hordeum bulbosum* with wheat (*Triticum aestivum* L.) can also induce haploid generation and has become an important method in wheat breeding (Kasha and Kao, 1970). Li et al. (2017),studied the whole-genome sequencing of pollen donor grains and sequencing of induced embryos and endosperm revealing that continuous chromosomal fragmentation during gametophyte meiosis causes embryonic haploidogenesis. Moreover, the specific elimination of paternal chromosomes during embryonic development is the primary reason for haploid induction reported in maize and wheat (Gernand et al., 2005; Zhao et al., 2013). Indeed, haploid induction in maize has been confirmed to be related to two genes, *PHOSPHOLIPASE A1* (*ZmPLA1)* and DOMAIN OF UNKNOWN FUNCTION 679 MEMBRANE PROTEIN (*ZmDMP)*. A 4-bp (CGAG) insertion in *ZmPLA1* may cause frameshift mutations that leads to the early termination of protein translation and triggers maize haploid induction (Liu et al., 2017). *ZmPLA1* is localized in the cytoplasm of sperm cells. Mutant pollen showed differentially expressed genes, including mediated pollen tube guidance genes, sperm capacitation genes, genes associated with calcium ion signaling during fertilization and other processes, and genes that control protein synthesis such as inner membrane transport and signal recognition (Kelliher et al., 2017). The *ZmDMP* gene encodes the DUF679 domain protein, expressed in mature pollen and located in the cell membrane. The substitution of thymine (T) at the 131 bp from ATG start codon with cytosine (C) leads to a missense mutation (Zhong et al., 2019), although the precise mechanism remains unclear. Overall, the use of inducer line hybridization to trigger haploid induction is straightforward and is applied on a large scale. For example, since the discovery of the Stock-6 haploid induction line, induction line breeding has become a key technology in commercial maize breeding (Cai et al., 2012).

Fu et al. (2018) used interspecific hybridization and genome doubling techniques to breed two synthetic octoploid rapeseed (*Brassica napus* L.) lines, Y3560 and Y3380 (AAAACCCC, 2n = 8x ≈ 76), and discovered their high efficiency in haploid induction. When used as pollen donors, the offspring are maternal di-haploid rapeseed, with almost all genetic information originating from the female parent, and the induction rate ranges from 34.09% to 98.66%, accompanied by a small amount of paternal fragment infiltration. These octaploid rapeseed lines were named as the rapeseed double haploid inducer line. However, the inducer line is genetically unstable, and its self-inbred rapeseed isolates have different ploidies (Yin et al., 2020). The CHG methylation pattern is altered in the pollen (Yin et al., 2021). The induction ability of an induction line does not entirely depend on ploidy, and both ploidy and genotype determine the high induction efficiency (Luo et al., 2021). There is an interaction between the induction line and the maternal karyotype and cytoplasmic genotype (Zhang et al., 2021), and the induction can simultaneously breed the pol-sterile and the maintainer lines (Zhang et al., 2022). These induction lines have the potential to induce rapeseed and cruciferous vegetables to produce di-haploids (Li et al., 2021; Yang et al., 2022), providing new ideas for the expansion genetic resources of rapeseed and cruciferous vegetable.

Similar to maize haploid induction and *Arabidopsis EXPRESSED CENTROMERE SPECIFIC HISTONE* 3 (CENH3)-mediated haploid induction, the gene editing vector is introduced into the inducer line. By the induction, the maternal gene editing and loss of editing vector are quickly furnished in one step, and the editing efficiency can reach more than 20% (Li et al., 2021). Although this inducer line has been widely used in rapeseed and cruciferous vegetables (Yang et al., 2022), its induction mechanism is unclear, and it is not yet known whether its mechanism is the same as that of the maize and wheat-specific elimination of paternal chromosomes during embryonic development.

This study aimed to backcross the exogenous gene *cp4-EPSPS* into Y3380 as a marker gene and analyze the presence or absence of marker genes in the embryonic development of hybrid offspring to elucidate the mechanism of rapeseed doubled haploid induction and to lay a theoretical foundation for the application of rapeseed inducer lines in breeding.

## 2 Materials and methods

### 2.1 Plants materials and cultivation conditions

The primary test materials and hybrid combinations are shown in **Table 1**, and the number of chromosomes and genotypes are shown in **Supplementary Table S1**. The conventional variety, ZS11 (*B. napus* L.), was provided by the Oil Crop Research Institute of the Chinese Academy of Agricultural Sciences, whereas the remaining materials were provided by the Chengdu Academy of Agriculture and Forestry Sciences. Y3380 was synthesized and bred by Shaohong Fu of the Chengdu Academy of Agriculture and Forestry Sciences (**Supplementary Figure S1**). Y3380*-cp4* is a hexaploid rapeseed double haploid inducer containing the exogenous anti-glyphosate gene *cp4-EPSPS*, which was derived through multi-generation backcrossing (BC_3_F_6_) of Y3380 and tetraploid rapeseed 4122*-cp4* (*B. napus* L., containing FMV 35S+*cp4-EPSPS*). The *cp4-EPSPS* was detected using *cp4* test strips purchased from the Transgenic Testing Center of the Ministry of Agriculture, Oil Crop Research Institute of the Chinese Academy of Agricultural Sciences. Because Y3380*-cp4* is the only doubled haploid induction inducer used in this study, Y3380*-cp4* is abbreviated as Y3380. In addition, 4122 (*B. napus* L.), a transgenetic tetraploid variety generated by microspore culture techniques containing FMV 35S + *cp4-EPSPS*, was imported from University of Guelph in Canada.

**Table 1:** Hybrid combinations.

Maternal parent	Paternal parent	Note
3496	Y3380	For induction
3496	4122	positive control in *cp4-EPSPS tracking*
3496	3737	positive control in *cp4-EPSPS tracking*
4120	Y3380	For induction
4120	4122	positive control in *cp4-EPSPS tracking*
4120	ZS11	positive control in *cp4-EPSPS tracking*
0068A	Y3380	For induction
0068A	4122	positive control in *cp4-EPSPS tracking*
0068A	ZS11	positive control in *cp4-EPSPS tracking*

This study used three types of maternal materials with specific effects. 4120 is a hybrid F_1_ generation of the homozygous yellow leaf-dominant variety 6170 (*B. napus* L.) and homozygous variety ZS11 (*B. napus* L.). This was used as a homozygous female (*B. napus* L.) with dominant yellow leaf traits and was used to observe morphological characteristics; 3737 (*B. napus* L.) is *cp4-EPSPS* negative. 3496 was used as female (*Brassica juncea*, AABB) and was used for fluorescence *in situ* hybridization (FISH). 0068A is a *pol* CMS(cytoplasmic male sterile line) variety (*B. napus* L.) and was used to exclude induced F_1_ as a condition in which the maternal materials were not emasculated completely. All three females are *cp4-EPSPS* negative. The rapeseed materials were planted in the rapeseed planting base of Chengdu Academy of Agriculture and Forestry Sciences, Yangma, Chongzhou in September 2018 and managed according to the conventional way of rapeseed cultivation. We completed the hybridization in March 2019. In preparation for pollination, we removed the young buds of 4120 and 3496 that were about to flower, selected the buds that had bloomed after 2–3 d, emasculated them manually, and bagged them. We bagged the inflorescences of 0068A before blooming, and 2–3 d after emasculation of females, the flowers of females were artificially pollinated with the paternal rapeseed pollen and bagged. The pollinations were performed in the field plants in Yangma, Chongzhou.

### 2.2 Rapeseed embryo fertilization observation

We took the siliques from all combinations from 4120 and 0068A in **Table 1** for observation at 48 h after pollination and fixed them with Carnoy’s solution (ethanol:glacial acetic acid = 3:1) for more than 24 h. Then, we transferred them to 70% ethanol for 30 min, rinsed them with distilled water for 30 min, and transferred them to 1 M NaOH solution for softening treatment for 12 h. Then, we took them out and rinsed them with distilled water three times, 10 min each time. We used 2% modified aniline blue dye solution to stain the siliques in complete darkness for 3–5 h (Takeuchi and Higashiyama, 2016). We placed the stained siliques on a glass slide, removed the silique wall with tweezers and a dissecting needle, covered them with a cover glass and gently pressed the slide, then took pictures under the ultraviolet light of a fluorescence microscope to observe the intensity of fluorescence on the *Brassica* stigma. We counted the fertilization rate 48 h after pollination. Fertilization rate percentage was calculated by the following formula: fertilization rate % = the number of ovules with fluorescent dots in the silique/total number of ovules in the silique × 100 (Zhou et al., 2022).

### 2.3 Observation dynamic change of embryonic development

We picked one to two siliques from all combinations in **Table 1** for observation every few days after pollination, except the siliques from combination 3464 × 3737, and dissected the rapeseed seeds under the stereomicroscope (S8AP0, LEICA). After photographing the siliques and seed development by SLR camera (E0S 200D, Cannon) and asana microscope, we conducted statistical analysis of the seed mortality and survival rates. We recorded the daily temperature changes in the field every day after pollination. We then used Microsoft Office Excel 2007 and IBM SASS Statistics 21 to count and analyze the correlation between seed mortality and temperature in different time periods.

After the siliques were ripened, we counted the effective silique rate and the average effective fruiting number of siliques: effective silique rate = number of available siliques/number of pollinated siliques × 100%; average effective fruiting number = total number of induced F_1_ seeds/number of effective siliques.

### 2.4 Marker gene tracking

According to the observation of seed development during the dissection of induced F_1_ seeds, the period for detecting the F_1_ seed marker gene using the *cp4* rapid test strip and the amplification time point for direct PCR of induced F_1_ seeds were proposed. A marker gene-tracking test was performed to determine whether the marker gene was integrated into the induced F_1_, whether it was lost after integration, and whether the probability of containing the marker gene in the seed development process changed according to the proposed period and time point. We used the *cp4-EPSPS* gene rapid test strip to determine whether the parents and induced F_1_ contained the *cp4-EPSPS* gene. The *cp4* rapid test strips were purchased from the Transgenic Testing Center of the Ministry of Agriculture, Institute of Oilseeds of the Chinese Academy of Agricultural Sciences. We collected a small amount of clean and fresh rape leaves, siliques, and other tissues; placed them in a 1.5-mL centrifuge tube; and avoided cross-contamination. Then, we added 500–1,000 μL deionized water to the centrifuge tube, mashed the leaves with a toothpick until the liquid turned green, and picked out solid impurities. We removed the *cp4* rapid test strip from the package as soon as possible and placed it in the sample supernatant of the centrifuge tube in the direction of the arrow. After the background color of the test strip faded within 5–10 min, a positive signal was observed to determine whether the strip contained transgenic ingredients.

Anatomical observations of the developmental process of the induced F_1_ seeds showed that the developmental process was accompanied by seed death. Therefore, when tracking the *cp4-EPSPS* gene, it was necessary to investigate its presence or absence of *cp4-EPSPS* in both live and dead seeds. We used the 24th day as the dividing line between early and late stage, and took one to five siliques at a certain number of days after pollination, one to two siliques in the early stage, and three to five siliques in the later stage, then put them in the refrigerator at −80°C after liquid nitrogen flash freezing. We took a single seed, placed it in a 200-μL centrifuge tube, added 20–50 μL Extraction Solution, poked the embryo or small seed with a tip to soak all the samples into the solution, then treated them with a water bath at 95°C for 10 min, added an equal amount of ddH_2_O, and mixed well. Samples could be left at 4°C or −20°C for standby or immediately for PCR amplification. *cp4-EPSPS* gene is derived from bacteria. In order to exclude the interference of bacteria containing *cp4-EPSPS* gene in the environment, we used the *FMV 35S* (Figwort mosaic virus) promoter closely linked to *cp4-EPSPS* gene instead to detect the presence of inducer line marker genes in the process of induced F_1_ seed growth by direct PCR. The positive control seeds and induced F_1_ seeds were subjected to PCR amplification of *FMV 35S* promoters. In this experiment, primers including primers of the *FMV 35S* promoter of the foreign gene *cp4-EPSPS* (Tian et al., 2009) and primers of the rapeseed internal reference gene *β-actin* (Pan et al., 2001) were used, and the length of the proposed amplification fragments was 365 bp and 322 bp, respectively (**Supplementary Table S2**). PCR amplification procedure was as follows: 94°C, 4 min; 94°C, 30 s; 56°C, 30 s; 72°C, 2 min (Goto Step2, 35 cycles); 72°C, 10 min; and 4°C to keep warm. We sent the PCR amplification products to Qingke Biotechnology Co., Ltd. for sequencing and compared the results with the known *FMV 35S* promoter sequences from NCBI. The results showed that the sequenced DNA sequence length was 365 bp, which was the same length as the known sequence, and the similarity rate was 99% (**Supplementary Figure S2**), indicating that the *FMV 35S* promoter primer could be used for this experiment and PCR amplification detection. The remaining PCR products were analyzed using 1% agarose gel electrophoresis. The gel imaging analysis was imaged using a gel imager (SYDR2/1635 Genegenius). We used Microsoft Office Excel 2007 and IBM SASS Statistics 21 to analyze the correlation between the rate of induced F_1_ containing marker gene and seed mortality and the relationship between marker gene elimination rate and temperature in the same time period.

### 2.5 Embryonic cp4 immunofluorescence

We took the siliques of F_1_ from 0068A every few days after 3 days of pollination, put them into 4% sodium hypochlorite solution for disinfection for 10 min, washed them three times with sterile water, then removed the ovules with a dissection needle and forceps on the sterile operation table, fixed them with paraformaldehyde for 24 h, and stored them in the refrigerator at −4°C for later use. After dehydration, the ovules were embedded in Surgipath (Leica) Fsc22 cryo-embedding agent and placed below −30°C for freeze coagulation. We cut the ovules into sections of approximately 5–10 nm with cryomicrotome (Leica_CM1900), then dried them naturally with a glass slide stick. We circled the samples on the slide with a waterproof pen and sealed the frozen section in 3% skim milk (0.1M phosphate-buffered saline (PBS), pH = 7.4) at room temperature for 1 h. We added primary antibody anti-*cp4* (1:1,000, produced by Beijing Protein Innovation Co., Ltd.), incubated at room temperature for 2 h, added 0.1 M PBS (pH = 7.4), washed three times, 5 min each, then added secondary antibody fluorescein isothiocyanate-conjugated goat anti-mouse IgG (1:100, produced by Beijing Protein Innovation Co., Ltd.), and incubated at room temperature protected from light for 1 h, washed with 0.1 M PBS (pH = 7.4) three times, 5 min each, and then placed the sections under the confocal microscope to observe the fluorescence signal.

### 2.6 FISH

Yin (Yin et al., 2021) used FISH to demonstrate that only the A and C subgenome chromosomes were contained in the doubled haploid inducer line. Therefore, we performed FISH of induced F_1_ obtained by hybridization of doubled haploid inducer line Y3380 (AAAACC) with 3496 (*B. juncea*, AABB). A combination of 3496 × 4122 was used as the positive control, and 3496 inbred were used as the negative control to determine whether there were large fragments of paternal chromosomes in induced F_1_. We germinated black mustard (*Brassica nigra*, BB) seeds containing the B genome in sterilized earthen pots, grew them in an incubator at 25°C, and took young leaves for DNA extraction. We extracted black mustard seeds’ DNA using the FastPure Plant DNA Isolation Mini Kit’s according to the manufacturer’s instructions. The special DNA probe BNIH123L05 (Xiong and Pires, 2011) in the BAC library containing the specific *Brassica* C genome was provided by Huazhong Agricultural University and was introduced into the *Escherichia coli* plasmid vector. The bacterial solution of *E. coli* was thawed and activated in LB medium (25 µg/mL chloramphenicol) and cultured at 37°C. We shook the bacteria overnight and scribed them to an LB medium plate (25 µg/mL chloramphenicol) and cultured them overnight at 37°C. A single colony of *E. coli* was selected in liquid LB medium (25 µg/mL chloramphenicol) and cultured overnight at 37°C. The DNA probe of the overnight culture broth was extracted according to the instructions of the Gold HiEndofree Plasmid Maxi Kit’s instruction method to extract the probe DNA of the specific *Brassica* C genome sequence BNIH123L05 from the BAC library. The probe was labeled using the Nick Translation method (Knytl et al., 2013). The fluorescein-12-d UTP marks the B genome probe in green, and the Tamra-CTP marks the C genome probe in red. Fluorescently labeled probes were used as hybrid probes. Rapeseed was disinfected in 75% alcohol for 30 s at 9 a.m., sterilized them with 0.1% mercury for 5 min, and rinsed them with sterilized water more than three times. Then, the seeds were placed in sterilized flasks and incubated darkly at 25°C for 24 h in the dark. The flasks were placed in the incubator at 9 a.m. the next day for 12 h at 15°C. At 9 p.m., the temperature was set to 25°C in the incubator, and the flasks were incubated for 12 h. At 9 a.m., on the third day, we took the root tips in the clean bench, placed them in 0.002 M 8-hydroxyquinoline for 2–2.5 h, fixed them with Carnoy’s solution (ethanol:glacial acetic acid = 3:1) for 4 h, transferred them to 70% alcohol, and stored them in the refrigerator at −20°C. We took the fixed root tips, washed them three times with distilled water, cut the root tip meristem zones, absorbed the water, and put them in 30 μL enzymatic hydrolysate (1% pectinase, pectolyase Y-23; 2% cellulase, cellulase Onozuka R-10) enzymatically hydrolyzed at 37°C for 40 min. They were rinsed twice with distilled water, then transferred in 0.075 M KCl for 30 min, and rinsed twice with 70% ethanol. A small amount of liquid was left in the centrifuge tube for the last rinse, and the root tips was mashed with a grinding rod and centrifuged at 5,000 r/min for 3 min. Then, we discarded the supernatant, added 20 μL glacial acetic acid to suspend the pellet, and dripped slide glasses in a slide box with a humidity >50%. After the slide glass was dry, 15 μL Carnoy’s solution (ethanol:glacial acetic acid = 3:1) was added for fixation. We allowed the slide glass to dry and put them in the slide box. We placed the slide glass in a UV cross-linker for cross-linking (125 mJ/cm^2^) twice and then dropped 10 µL hybridization solution (Fu et al., 2018) (labeled probe 1 µL + 4.5 µL 2SSC + 4.5 µL × TE) on each slide and covered with an over glass. We put it in a metal humid box with moist paper towels, heated and denatured in boiling water for 5 min, then hybridized in a thermostat at 42°C for 14–16 h. After the hybridization was completed, we put the slide glass in a 2 × SSC preheated overnight at 42°C to wash off the cover glass, then dried the slide glass, dropped 15 µL DAPI (containing antifading agent), and placed it on the fluorescence microscopeg. Then, image acquisition was carried out. If the offspring contain the same fluorescence signal from the C genome chromosome as Y3380, the seeds contain the paternal chromosomes.

### 2.7 Plant morphological characteristics observation

We planted the F_1_ generation in the open field in the rape-planting base of Chengdu Academy of Agriculture and Forestry Sciences, Yangma, Chongzhou in September 2019 and used an SLR camera to photograph phenotypes of females, doubled haploid inducer line, and hybrid offspring, and observed the morphological characteristics including leaf colors, leaf types, and leaf lobes of parents and induced F_1_.

### 2.8 Flow cytometry to identify ploidy

Flow cytometry has been widely used in the determination of DNA content (Crowley et al., 2016; Ureta et al., 2017). In this experiment, flow cytometry was used to determine the DNA content of the parents, F_1_, and induced F_1_ to determine the ploidy according to the reference. We took fresh young leaves of the tested plants from 8 to 11 a.m., washed them with distilled water, and dried them with filter paper. Then, we avoided the main leaf veins, took the leaves with a hole punch with a diameter of 8 mm, placed them in a pre-cooled Petri dish, and added 0.5 mL of pre-cooled cell lysis buffer (15 mM Tris, 2 mM disodium oxalate, 0.5 mM spermine tetrachloride, 80 mM potassium chloride, 20 mM sodium chloride, 0.1% Triton-100, and 15 mM β-mercapto ethanol, pH = 7.5, filtered with 0.22 mM filter membrane). We used a sharp razor to chop the leaves quickly to tiny particles and filtered with a 300-mesh filter into a 2-mL EP tube. After that, 1.5 mL PI staining solution (5% propidium iodide and 5% RNase) was added in the dark for 30 min. Then, we measured the progressed materials on the flow cytometer (Accuri C6 Plus, BD Biosciences, Franklin Lakes, NJ, USA) till the flow cytometer collected 20,000 cell particles. The PI-stained cell nucleus suspension entered the flow chamber and emitted fluorescence through laser irradiation. Then, BD AccuriC6 Plus was used to determine the fluorescence value (peak value) at the position of cell division G1. The G1 peak of the tetraploidy parent was used as a reference to determine the ploidy of the offspring. Sample ploidy = reference material ploidy × (fluorescence value (peak value) at the position of sample G1 peak/fluorescence value (peak value) at the position of G1 peak of reference material). The parental materials were used as controls.

### 2.9 SNP loci chip analysis

SNP chip detection was completed by Wuhan Shuanglvyuan Innovation Technology Research Institute Co., Ltd., using *Brassica* 50K SNP (single nucleotide polymorphism) chip (developed jointly by Wuhan Shuanglvyuan Innovation Technology Research Institute Co., Ltd. and Huazhong Agricultural University, professionally customized by Illumina Infinium) to identify rapeseed materials. Illumina Genome Studio software was used to analyze and screen the SNP raw data, and the SNP loci, which have markers with AA or BB genotype frequency of 0, minimum genotype frequency <0.05, SNP yield <0.80, and deletion rate >5%, were removed. SNP loci deletion rate, heterozygosity rate, and minimum allele frequency were calculated using perl programming, and all SNP markers were statistically performed. SNP probe sequences were compared to the latest reference genome (*B. napus*, v2) of *B. napus* L. using BowTie software (Langmead et al., 2009) to obtain SNP loci that uniquely matched the genome. Joining cluster method of Tassel5 software was used to calculate the Nei genetic distance and construct genetic cluster map. The PI_Hat coefficient was calculated using Plink software 24 (Purcell et al., 2007). The polymorphism markers between parents were used as analytical objects to detect the penetration of the paternal genome in the corresponding offspring, and the rate of paternal loci penetration in offspring was calculated: penetration rate of paternal SNP loci in induced F_1_ = number of SNP loci consistent with males in induced F_1_/(number of SNP loci consistent with male in induced F_1_ + number of SNP loci consistent with female in induced F_1_) × 100%.

## 3 Results

### 3.1 Observation of fertilization rate, F_1_ seeds formation process, and morphological characteristic

**Figure 1:**
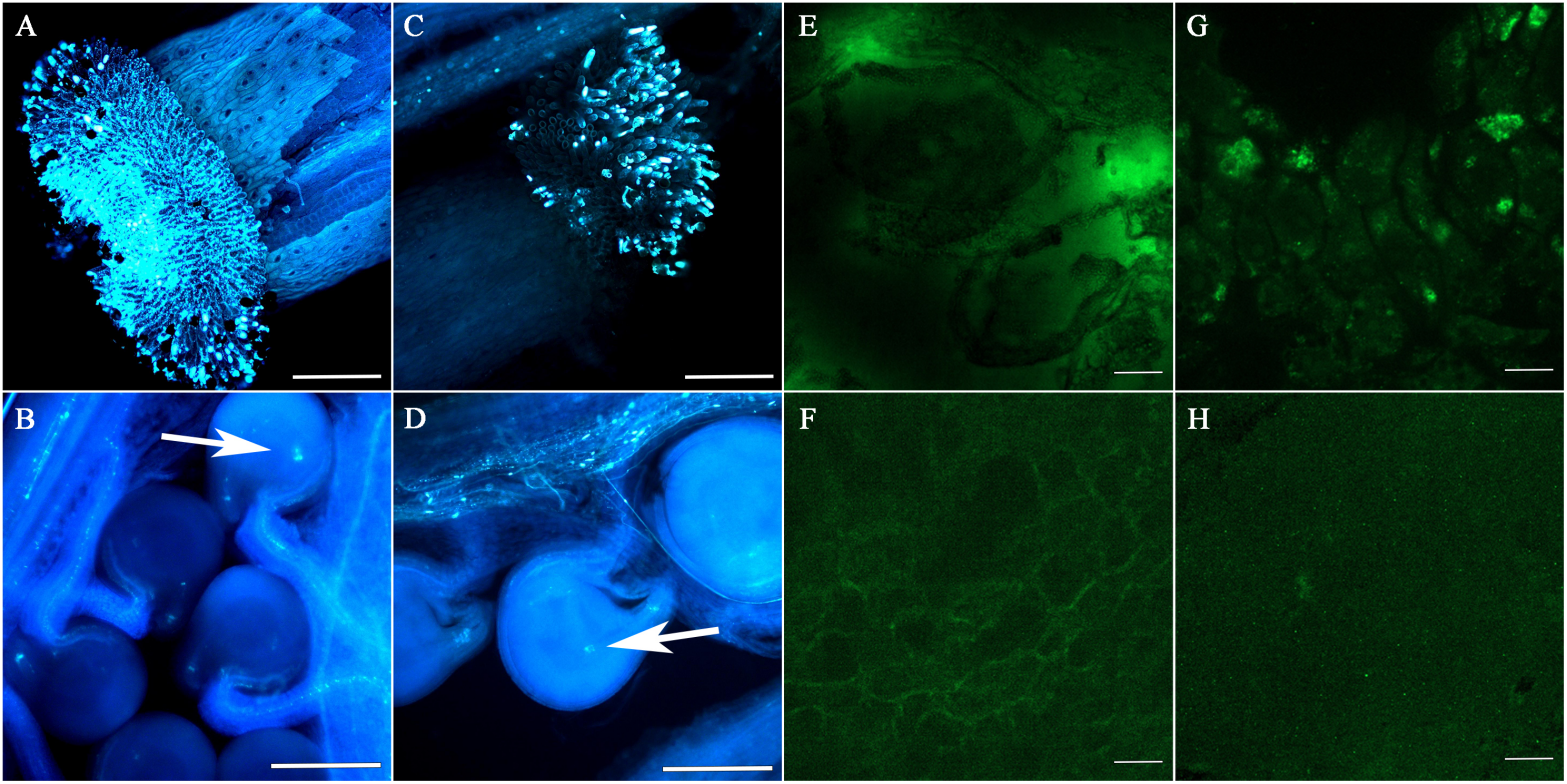
The adhesion of pollen by the stigmas of each hybrid combination after 24 h of pollination and the extension of pollen tubes in the siliques after 48 h of pollination, and immunofluorescence localization of *cp4-EPSPS* in embryos. **(A)** The pollen of Y3380 played a role on the stigma of 4120. **(B)** The extension of the pollen tube in the silique after the pollen of Y3380 played a role on the stigma of 4120 for 48 h; the arrow indicates that the pollen tube entered through micropyle and complete fertilization. **(C)** The pollen of Y3380 played a role on the stigma of 0068A. **(D)** The extension of the pollen tube in the silique after the pollen of Y3380 played a role on the stigma of 0068A for 48 h; the arrow indicates that the pollen tube entered through micropyle and complete fertilization. **(A–D)** Bar = 1 mm. **(E)** Combination 0068A × Y3380 on the third day after pollination, containing green fluorescence (big green highlights). **(F)** Negative control combination 0068A × ZS11, without green fluorescence. **(G)** Positive control combination 0068A × 4122, containing green fluorescence. **(H)** Combination 0068A × Y3380 on the 22th day after pollination, without green fluorescence (big green highlights). **(E–H)** Bar = 100 μm.

**Figure 2:**
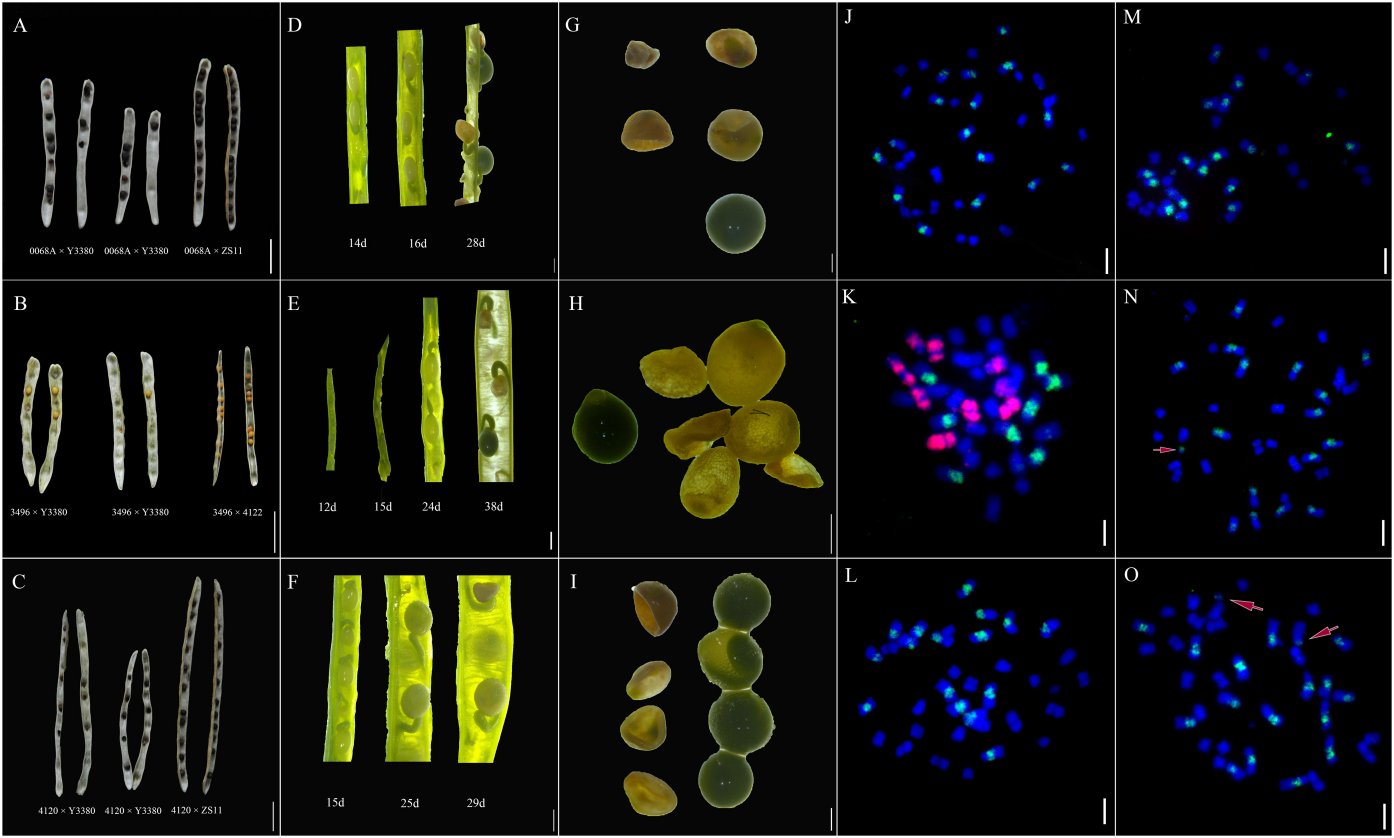
Development of siliques and seeds of induced F_1_ and FISH diagrams of induced F_1_ of 3496 and F_1_ of control combinations. **(A)** Mature siliques and seeds of combinations 0068A × Y3380 and 0068A × ZS11. **(B)** Mature siliques and seeds of combinations 3496 × Y3380 and 3496 × 4122. **(C)** Mature siliques and seeds of combinations 4120 × Y3380 and 4120 × ZS11. **(A–C)** Bar = 1 cm. **(D)** Siliques of combination 0068A × Y3380 on the 14th, 16th, and 18th day after pollination. **(E)** Siliques of combination 3496 × Y3380 on the 12th, 15th, 24th, and 38th day after pollination. **(F)** Siliques of combination 4120 × Y3380 on the 15th, 25th, and 29th day after pollination. **(G)** Seeds of combination 0068A × Y3380 on the 34th day after pollination. **(H)** Seeds of combination 3496 × Y3380 on the 42th day after pollination. **(I)** Seeds of combination 4120 × Y3380 on the 33th day after pollination. **(D–I)** Bar = 1 mm. **(J)** FISH diagram of negative control 3496 self-pollination. **(K)** FISH diagram of F_1_ positive control combination 3496 × 4122. **(L–O)** FISH diagram of induced F_1_ of combination 3496 × Y3380. **(J–O)** Bar = 20 μm. The B genome chromosomes appear as green, the A genome chromosomes appear as blue, and the C genome chromosomes appear as red.

To study the development of F_1_ seeds, we observed the fertilization and the developmental status of F_1_ siliques and seeds at different developmental stages following pollination. Pollen adhesion of the 4120 × Y3380 and 0068A × Y3380 combinations was observed at 24 h after pollination. The pollen of Y3380 had strong fluorescence intensity on the stigma of 4120 and 0068A (**Figures 1A, C**) and adhered well to the stigma. After 48 h of pollination, many pollen tubes entered the ovule, and conspicuous fluorescence signals appeared at the center of the ovule, indicating that the ovule had been fertilized (**Figures 1B, D**). The fertilization rates for the two combinations reached 97.92% and 98.72%. During the ripening period, we observed the appearance of the siliques. We noted that the lengths of the siliques of F_1_ of combinations 4120 × Y3380, 0068A × Y3380, and 3496 × Y3380 were slightly shorter than those of the control combinations 4120 × ZS11, 0068A × ZS11, and 3496 × 4122. The transverse diameter of the siliques was also different and included dead seeds within them (**Figures 2A–C**). We counted all the siliques of the combinations 4120×Y3380, 0068A × Y3380, and 3496 × Y338 together. Approximately 20%–40% of the ovules in the siliques did not expand or grow, eventually dried up and died 15 d after pollination (**Figures 2D–F**). At 23–27 d after pollination, 40%–80% of the seeds developed abnormally. They initially grew and swelled, then collapsed, wrinkled, and died, while the remaining seeds grew and swelled normally with tender green seed coats and were full and shiny in appearances (**Figures 2A–C**). At 29 d after pollination until seed maturity, 80%–90% of the seed coats changed from milky white to brown and tarnished, and the seeds became dry and flat (**Figures 2G–I**), and only one to five mature seeds developed normally.

**Figure 3:**
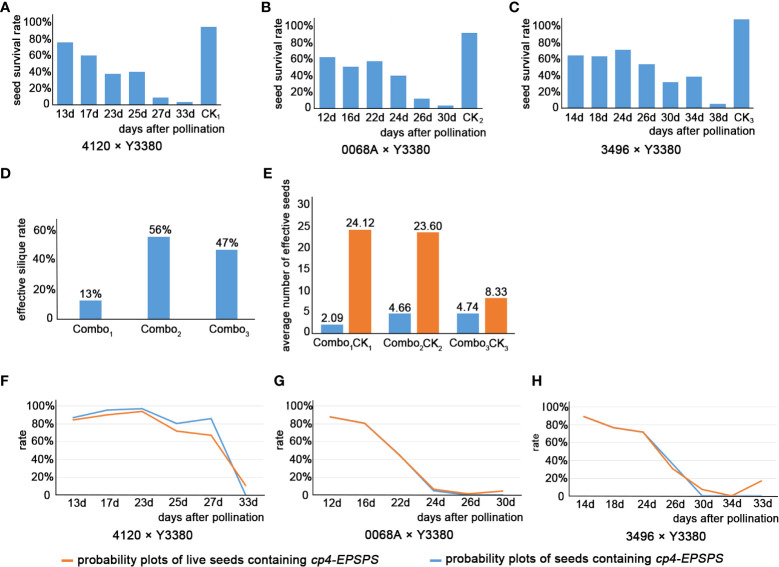
Histogram of survival rate of induced F_1_ seeds, histogram of effective silique rate, and histogram of average number of induced F_1_ effective seeds, and the probability diagrams of induced F_1_ seeds containing *cp4-EPSPS* of three combinations. **(A–C)** Histogram of survival rate of induced F_1_ seeds. **(D)** Histogram of effective silique rate of induced F_1_. **(E)** Histogram of average number of induced F_1_ effective seeds. **(A–E)** Combo_1_ represented combination 4120 × Y3380, Combo_2_ represented combination 0068A × Y3380, Combo_3_ represented combination 3496 × Y3380, CK_1_ represented combination 4120×ZS11, CK_2_ represented combination 0068A × ZS11, and CK_3_ represented combination 3496 × 4122. **(F)** Probability diagrams of induced F_1_ seeds containing *cp4-EPSPS* of combination 0068A×Y3380. **(G)** Probability diagrams of induced F_1_ seeds containing *cp4-EPSPS* of combination 3469×Y3380. **(H)** Probability diagrams of induced F_1_ seeds containing *cp4-EPSPS* of combination 4120×Y3380.

The results showed that after pollinating the females with the pollen of the inducer line, there was an abnormality in the induced F_1_ fertilization or embryonic development. While observing the developmental morphology of the induced F_1_ seeds, the mortality and survival rates of seeds at different periods were calculated (**Supplementary Table S3**). The day temperature after pollination was recorded in **Supplementary Table S4**. We found that the survival rates of the seeds decreased significantly after pollination for 23 d until maturity (**Figures 3A–C**), whereas the temperature increased faster in the late stage of seed growth, and there was a time difference between the three maternal pollinations. To verify whether the mortality rate of induced F_1_ seed correlated with temperature changes, we performed a correlation analysis (**Table 2**). The results showed a low correlation between the average temperature changes and the changes in the mortality rate of induced F_1_ seeds during development. Thus, seed death during development appears independent of the ambient temperature after pollination. The effective silique rate and effective fruiting number were also investigated in the induced F_1_ seeds. The effective silique rates of F_1_ of combinations 4120 × Y3380, 0068A × Y3380, and 3496 × Y3380 were 13%, 56%, and 47%, respectively (**Figure 3D**). The average effective fruiting numbers were 2.09, 4.66, and 4.74, respectively (**Figure 3E**). For the control combinations, the effective silique rates of F_1_ of 4120 × ZS11, 0068A × ZS11, and 3496 × 4122 were all 100%. The average effective fruiting numbers were 24.12, 23.60, and 8.33, respectively. The effective silique rate and effective fruiting number of the induced F_1_ from the three females were lower than those of the control combination of the same females. The F_1_ of the combination 4120 × Y3380 had a three- to fourfold difference in the effective silique rate and a twofold difference in the average effective fruiting number compared with the other two combinations, indicating that the effective silique rate and effective fruiting number of the induced F_1_ may be related to the maternal genotype.

**Table 2:** *cp4-EPSPS* gene strip detection of induced F_1_, correlation between the average temperature change of induced F_1_ seeds and the changes of seed mortality in different time periods, correlation between the average temperature change and seed mortality in F_1_ seeds, and the probability of seed containing *cp4-EPSPS* genes in different time periods.

Combination	Number of plants tested	Number of *cp4* positive plants	Positive occupancy ratio	r_t&_ *_cp4_*	r_m&_ *_cp4_*	r_t&m_
4120 × Y3380	5	2	40.0%	0.6219	−0.7704	0.1209
0068A × Y3380	25	0	0%	−0.4697	−0.8126	0.0736
3496 × Y3380	13	0	0%	−0.0848	−0.7770	0.1439
Total	43	2	4.65%			

r_t&*cp4*_ represents the correlation between the average temperature change and the probability of seed containing cp4-EPSPS genes in different time periods. r_m&*cp4*_ represents the correlation between the seed mortality in F_1_ seeds and the probability of seed containing cp4-EPSPS genes in different time periods. r_t&m_ represents the correlation between the average temperature change in induced F_1_ seeds and the changes in seed mortality in different time periods.

### 3.2 Induced F_1_ marker gene tracking and embryonic cp4 immunofluorescence

**Figure 4:**
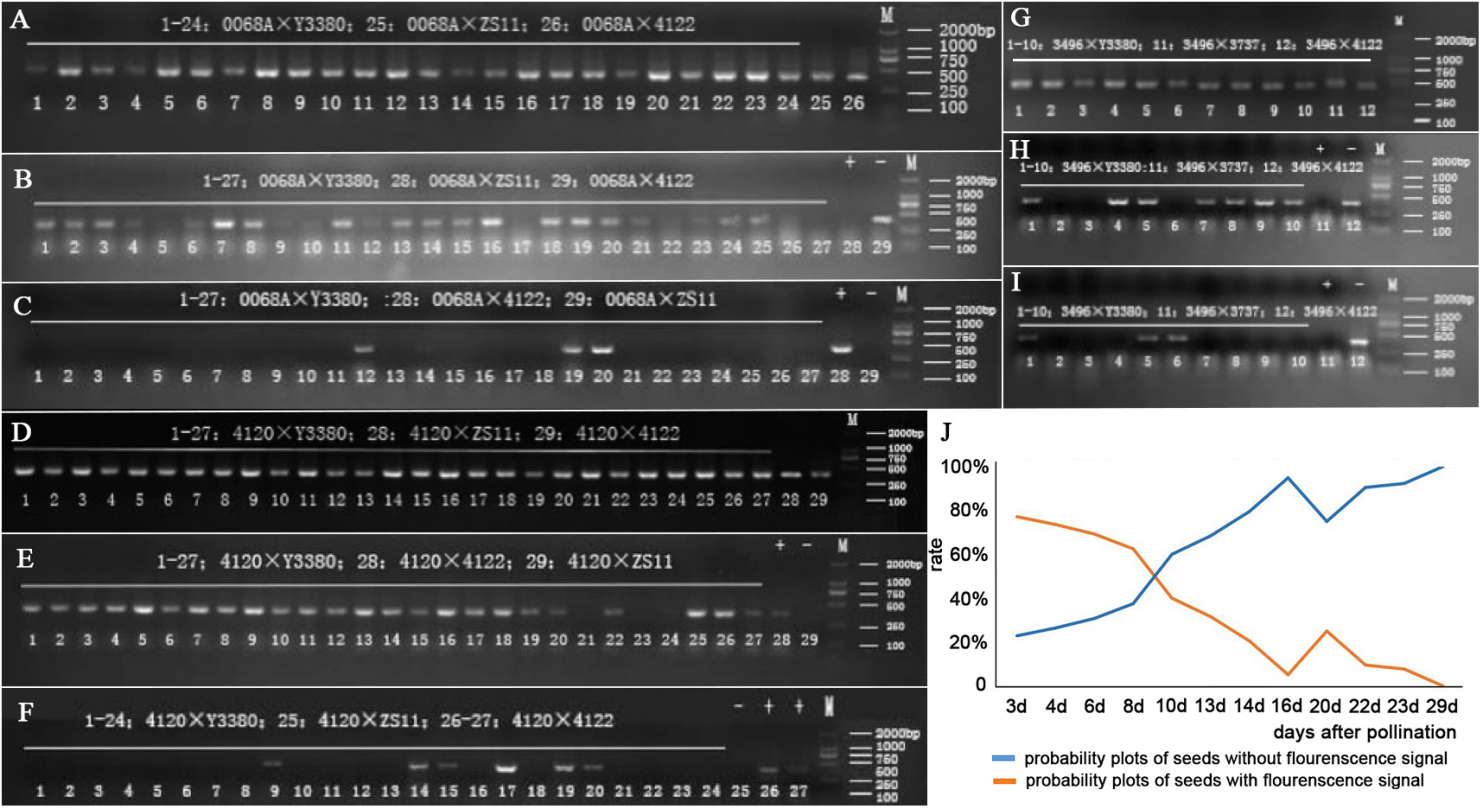
Internal reference genes and *cp4* gene *FMV35S* promoter PCR amplification electrophoresis of induced F_1_ of three combinations, and the probability of seeds of 0068A with and without fluorescent signals. **(A)** *cp4* gene *FMV35S* promoter PCR amplification electrophoresis of induced F_1_ of combination 0068A × Y3380. **(B, C)** PCR amplification of the *FMV35S* promoter on the 21st day and 29th day after pollination of induced F_1_ of combination 0068A × Y3380. **(D)** *cp4* gene *FMV35S* promoter PCR amplification electrophoresis of induced F_1_ of combination 4120 × Y3380. **(E, F)** PCR amplification of the *FMV35S* promoter on the 14th day and 24th day after pollination of induced F_1_ of combination 4120 × Y3380. **(G)** *cp4* gene *FMV35S* promoter PCR amplification electrophoresis of induced F_1_ of combination 3469 × Y3380. **(H, I)** PCR amplification of the *FMV35S* promoter on the 18th and 26th day after pollination of induced F_1_ of combination 3469 × Y3380. **(J)** The probability of seeds of 0068A with and without fluorescent signals.

After pollination, the young siliques were tested daily for *cp4-EPSPS* gene. Approximately 8 d post-pollination, positive results of *cp4-EPSPS* were detected in the F_1_ embryos of combinations 4120 × Y3380, 0068A × Y3380, and 3496 × Y3380 (**Supplementary Figure S2**). This indicated the successful integration of paternal pollen with maternal eggs and embryo development. However, the embryos were too small at 8 d post-pollination, and it was not until the 12th day that single embryos were suitable for the direct PCR detection of the marker gene. Statistical results are presented in **Supplementary Table S4**. The probability of F_1_ for combinations 4120 × Y3380, 0068A × Y3380, and 3496 × Y3380 containing *cp4-EPSPS* decreased during growth (**Figures 3F–H**; **Figures 4A–I**). At 13–23 d after pollination, the probability of F_1_ embryos containing *cp4-EPSPS* gene in combination with 4120 × Y3380 reached 97.27%, but then declined gradually to 0% at 23–33 d. The probability of the 0068A × Y3380 combination decreased the fastest at 12–24 d after pollination, from 87.31% to 6.31%, and gradually stabilized at 1%–4% at 24–30 d. The probability of combination 3496 × Y3380 at 14–24 d after pollination decreased slowly, from 88.89% to 71.43%, and the probability finally decreased to 0% at 24–34 d. Owing to higher seed death rates on the 38th day, 12 embryos were detected using PCR, and only two seeds contained the *cp4-EPSPS* gene, with a probability of 16.67%. At the maturity stage, the probability of seeds containing the *cp4-EPSPS* gene in all seeds that were detected from the three combinations was 0%–16.67%, the probability in live seeds decreased to 0%, and it was mainly detected in dying seeds. This suggests that paternal genes are eliminated during embryonic development We analyzed the correlation between the change in the probability of seeds containing the *cp4-EPSPS* gene in the induced F_1_ and the average temperature change during seed development at different periods (**Table 2**). The results showed that there were no significant correlations among these variables. Induced F_1_ embryos kept dying during growth, during which the embryo survival rate (**Figures 3F–H**) and the probability of embryos containing the *cp4-EPSPS* gene both decreased; therefore, the correlation analysis between embryo mortality and the probability of embryos containing the *cp4-EPSPS* gene was analyzed (**Table 2**). The results showed a positive correlation between the probability of embryos containing *CP4-EPSPS* gene and embryo mortality, indicating that embryo death was related to eliminating paternal genes or chromosomes during embryonic development. Based on the above results, the probability of embryos containing *cp4-EPSPS* gene and embryo survival or mortality rates also differed depending on the maternal genotype, Eliminating the inducer line’s gene might have obvious interaction effects with the maternal genotype (Zhang et al., 2021). To further verify that all marker genes in the embryos were eliminated in the induced F_1_, we germinated mature seeds of induced F_1_ to detect the presence of marker genes in a single seedling. Five seedlings were obtained from a combination 4120 × Y3380, of which two seedlings were detected to be positive (**Table 2**), proving that the induced F_1_ of 4120 had paternal gene penetration or parental hybridization. We randomly selected 25 and 13 seedlings from combinations 0068A × Y3380 and 3496 × Y3380, respectively, and the results were negative. This suggests that in induced F_1_, the fragments containing *cp4-EPSPS* gene of the inducer line differed from the maternal line and that only a small number of plants contained the *cp4-EPSPS* gene fragments, while most lost them did not.

We also performed *cp4-EPSPS* immunofluorescence localization on the 0068A × Y3380 combination embryos to further explore whether the marker gene continued to be specifically expressed during the elimination. Results showed that fluorescence signals were observed in embryos of the positive control combination 0068A × 4122 on different days (**Figure 1G**), indicating that *cp4-EPSPS* genes were expressed therein, In contrast, the negative control combination 0068A × ZS11 had no fluorescence signal (**Figure 1F**). At 3–23 days after pollination, fluorescence signals were observed in embryos of combination 0068A × Y3380 (**Figures 1E, H**), but with the increase in the day number, the number of embryos showing fluorescence signal gradually decreased (**Figure 4J**), the proportion decreased from 77.14% to 7.69%, Among the eight almost mature embryos harvested at 29 d after pollination, the proportion of fluorescent embryos decreased to 0%. These results indicated that paternal genes were continuously expressed during the process of elimination during embryonic development, which was consistent with the results of direct PCR detection of the marker gene and that the disappearance of the expression signal preceded the gene elimination.

### 3.3 Induced F_1_ FISH

Detection of the *cp4-EPSPS* marker gene in induced F_1_ embryos demonstrated that the genome of the inducer line was continuously being eliminated during embryonic development. However, fragments of the *cp4-EPSPS* gene were preserved in a very small number of the induced F_1_ offspring. To determine whether large fragments of the inducer line genome were preserved, we performed FISH detection of the root apical chromosome of the induced F_1_. The results indicated that the negative control 3496 self-inbred material contained 36 chromosomes, 20 A genome chromosomes (blue), and 16 B genome chromosomes (green) (**Figure 2J**). In contrast, the positive control interspecific hybrid F_1_ of the combination 3496 × 4122 had 37 chromosomes, 20 A genome chromosomes (blue), eight B genome chromosomes (green), and nine C genome chromosomes (red) (**Figure 2K**). The induced F_1_ offspring of combination 3496 × Y3380 only had A genome chromosomes (blue) and B genome chromosomes (green); no C genome chromosomes from the paternal inducer line were found, including both the induced F_1_ that meets the normal number of *B. juncea* chromosomes, 20 A genome chromosomes (blue), 16 B genome chromosomes (green) (**Figures 2L, M**), and the induced F_1_ that had 20 A genome chromosomes (blue) and 17 B genome chromosomes (green). In addition to the normal chromosome number, there were some small segments of the B genome (**Figure 2N**, chromosome pointed by arrow). There were also normal chromosomes, but there were 18–19 A genome chromosomes (blue) and16 B chromosomes (green), and one to two chromosomes had both the blue signal of the A genome and the green signal of the B genome (**Figure 2O**, the chromosome pointed by the arrow). These results from the marker gene tracking and the FISH observations of chromosomes suggest that the maternal egg and the paternal sperm formed zygotes. During the zygotic development, the chromosomes of Y3380 were selectively eliminated. This elimination process would also affect the replication of the maternal chromosome set.

### 3.4 Morphological observation and flow cytometric identification of induced F_1_ plants

**Figure 5:**
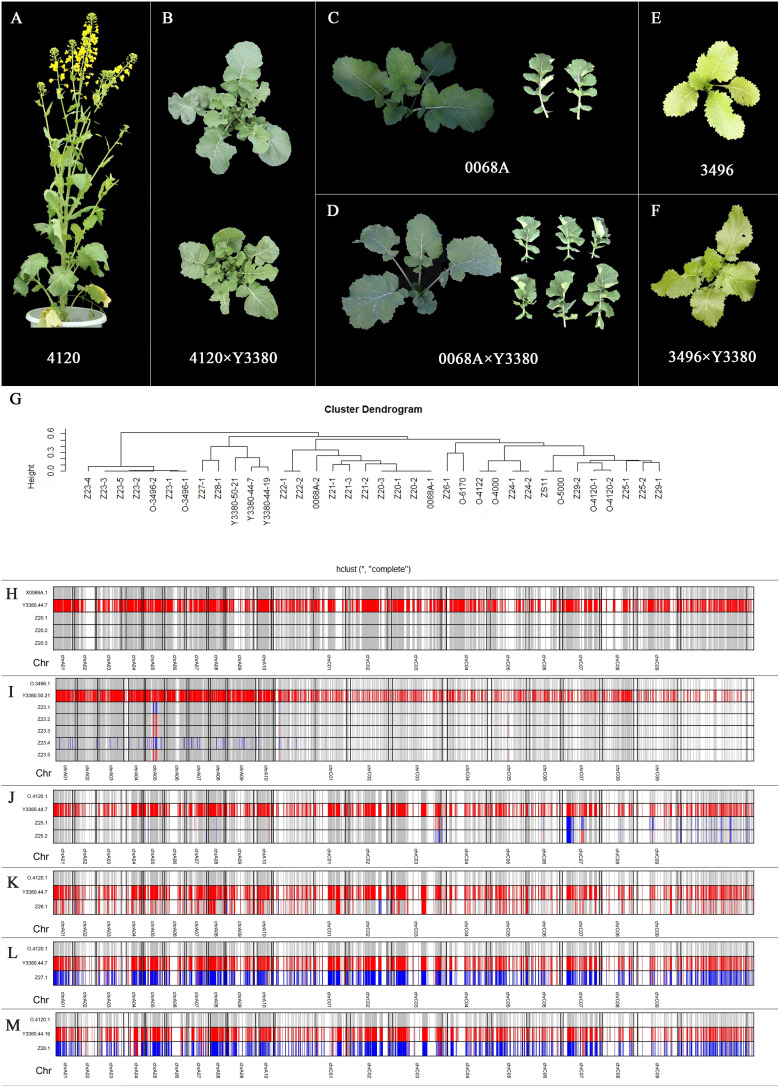
Plant phenotype of maternal materials and induced F_1_, flow cytometry results of induced F_1_, genetic clustering analysis, and genotyping diagram of parents and induced F_1_. **(A–F)** Plant phenotype of maternal materials and induced F_1_. **(G)** Genetic clustering analysis of parents and induced F_1_. **(H)** Genotyping diagram of 0068A, Y3380, and induced F_1_. **(I)** Genotyping diagram of 3496, Y3380, and induced F_1_. **(J–M)** Genotyping diagram of 4120, Y3380, and induced F_1_. **(G–M)** Z20-1, Z20-2, Z20-3, Z21-1, Z21-2, and Z21-3 represent induced F_1_ of combination 0068A × Y3380. Z22-1 and Z22-2 represent hybrid F_1_ of combination 0068A × ZS11. Z23-1, Z23-2, Z23-3, Z23-4, and Z23-5 represent induced F_1_ of combination 3469 × Y3380. Z24-1, Z24-2, Z24-3, and Z24-4 represent hybrid F_1_ of combination 3496 × 4122. Z25, Z26, Z27, and Z28 represent induced F_1_ of combination 4120 × Y3380. Z29 represent hybrid F_1_ of combination 4120 × ZS11. The longitudinal value is the number of each sample, the horizontal number indicates the chromosome corresponding to the segment, the colored part indicates that the parent has a different homozygous SNP site there, red indicates that the locus genotype is the same as Y3380, and gray indicates that the locus genotype is the same as the maternal material, and blue indicates that the genotype at that loci is heterozygous.

The traits of the induced F_1_ of 4120 were separated (**Figures 5A, B**), such as different leaf colors, which was green in induced F_1_ and yellow in heterozygous female 4120, different leaf types, and leaf lobes. Among the surviving offspring, single plants with increased leaf thickness might have been hybrid polyploid offspring. The phenotypes of the induced F_1_ of the 0068A × 3496 combination were highly similar to those of their maternal counterparts (**Figures 5C–F**). However, there were also obvious hybrid offspring in which two plants from the 0068A × Y3380 combination were similar to females, but the leaves were notably thickened and wrinkled, which might be hybrid polyploid offspring.

At the same time, the parents and F_1_ seedling stage were identified by flow cytometry, with the three tetraploids 4120, 0068A, and 3496 used as controls. The results of flow cytometry analysis can be seen in **Supplementary Table S5**. The G1 peak of hexaploid Y3380 was 625.8-689.9 D thousand lines, and the G1 peaks of tetraploids 4120, 0068A, and 3469 were 402.4-412.1 D, 340.6-357.0 D, and 413.8-469.4 thousand lines. We measured six plants of combination 4120 × Y3380, of which the G1 peak of four plants was 416.9-429.9 D thousand line and was judged to be tetraploid. The G1 of the other two plants were 495.9 and 523.9 D thousand lines (25% higher than the G1 peak of 4120) and were judged to be hybrid polyploids. The G1 peak of 88 plants of combination 0068A × Y3380 was 354.0-623.8 D thousand line, of which two plants were judged to be hybrid polyploids (50% higher than the G1 peak of 0068A), and the rest of the plants were judged to be tetraploid. The G1 peak of 21 plants of combination 3469 × Y3380 was 305.5-378.7 D thousand line and was judged to be tetraploid. The above results showed that a small number of parental hybrids and polyploidy appeared in the induced F_1_ offspring of 4120 and 0068A, while the majority of the progeny remained tetraploid, accounting for 96.52% of induced F_1_ progeny. The results were in agreement with the morphological observations.

### 3.5 Homozygous rate and genetic distance cluster analysis of induced F_1_ generation SNP loci

Morphological observation and flow cytometry can preliminarily determine the homozygosity of induced F_1_ plants. However, these methods are not accurate enough to detect the presence of chromosome fragments from the inducer line. Therefore, we selected tetraploid F_1_ that resembled the maternal parent and judged to be hybrid F_1_ by morphology observation and flow cytometry. Subsequently, we conducted analysis using a 50k SNP chip (**Supplementary Table S6**), and the results are summarized in **Supplementary Table S7**.

The homozygous rates of 0068A and ZS11 were Approximately 96%. Because these two parents are homozygous for more than 10 generations of backcrossing and self-inbreeding, we used SNP homozygous rate of approximately 96% as the criterion to confirm homozygous. The SNP homozygous rate of the inducer line Y3380 was 85.33%–89.06%, and Y3380 was a hexaploid rapeseed that was heterozygous for part loci. A total of six plants of combination 0068A × Y3380 were detected, among which the homozygous site rates of three strains were 96.61%–96.71%, which were homozygous, and the homozygous sites rate of the other three strains were 84.37%–87.20%, which were partially heterozygous. We detected two plants of 3469, in which homozygous rates were 96.17%–97.18%. Five plants of combination 3469 × Y3380 were detected. The four plants’ homozygous rates were 95.64%–97.10% and were judged to be homozygous. One plant had a homozygous rate of 90.58%, lower than the homozygous rate of the homozygous parent 3496, higher than Y3380, and was judged to be heterozygous for part loci. Two plants of 4120 were detected, and the homozygous rates were 96.17%–97.18%. A total of five plants of combination 4120 × Y3380 were detected. One plant had a homozygous rate of 95.89% and was judged to be homozygous, while two plants had homozygous rates of 84.06%–86.23%, and the average value was improved by 9.93% compared with 4120. It was identified as partially heterozygous tetraploid offspring based on flow cytometry results. The homozygous rates of two strains were 61.10% and 63.98%, and flow cytometry results revealed hybrid polyploid heterozygous offspring. This result indicates that the genotyping statistics of SNP markers can accurately determine the nature of induced F_1_ plants as homozygous, partially heterozygous or hybrids. The correlation between the homozygous loci rate of females and the probability of induced F_1_ containing *cp4*, the effective siliques rate of induced F_1_ seeds, and the average effective fruiting number were analyzed. The results showed that there was a very significant negative correlation between the homozygous loci rate of females and the probability of induced F_1_ containing *cp4* (p < 0.01, r = −0.9997**), and the homozygous loci rate of females was significantly positively correlated with the effective silique rate (p < 0.05, r = 0.9762*) and average effective fruiting number (p < 0.05, r = 0.9999*).

The genetic distance cluster of the samples was constructed by comparing the differences in SNP loci between the two materials (**Figure 5G**). Combined 0068A × Y3380, the homozygous offspring completely overlapped with the females, whereas the heterozygous offspring were slightly distantly distant from the females. In contrast, with the combination3496 × Y3380, the homozygous offspring were genetically close to the females, and heterozygous offspring were slightly further from females. However, in the case of the combination 4120 × Y3380, 4120 was a hybrid line with differences in egg cell genotypes, and the female 4120 and induced offspring were not well clustered together. The genetic distance between homozygous offspring and some heterozygous ones and 4120 was closer than that of Y3380, and the genetic distance between the heterozygous offspring and male Y3380 was closer than that of female 4120, which indicated that the maternally induced homozygous offspring and the hybrid offspring could be distinguished.

In the induced F_1_ of homozygous maternal 0068A and 3496, the paternal penetration rate of homozygous offspring was 0%–0.79%, and the maternal similarity rate was 96.13%–99.63%, and the paternal penetration rate of some heterozygous offspring was 2.86%–12.43%, and the maternal similarity rate was 71.91%–88.34%, respectively, indicating the presence of small fragments of paternal chromosome infiltration (**Supplementary Table S8**). In contrast, for heterozygous maternal 4120, the paternal penetration rate of homozygous offspring Z26-1 was 35.42% (**Supplementary Table S8**), indicating that in the process of zygote formation, the paternal chromosome elimination occurred before the genome doubling, and the genome of the Y3380 was not completely eliminated before doubling, becoming homozygous loci after doubling. Therefore, the SNP homozygous loci rate of Z26-1 was 95.89% (**Supplementary Table S8**), while the paternal penetration rates of some heterozygous offspring were 9.92% and 12.43%. Large paternal chromosome fragments penetration and hybridization occurred, and the paternal penetration rates of paternal hybrid polyploid heterozygous offspring were higher at 29.88% and 35.94%.

To understand the relationship between the offspring and parental genotype-specific loci more clearly, a genotype analysis of the offspring and parents was conducted, and the genotypes of the offspring at these loci were compared at homozygous loci where the parents were different. When the female was homozygous for 0068A, the homozygous offspring genotypes at the specific loci were almost identical to those of 0068A, which was consistent with the results of the gene penetration analysis (**Figure 5H**). When the female was homozygous for 3496, its homozygous offspring were prone to heterozygosity or exchanged with the Y3380 at some loci on chromosome A05. Some heterozygous offspring also contained the parental hybrid genotype loci on chromosomes other than A05 (**Figure 5I**). When the female was heterozygous at 4120, the homozygous offspring had different degrees of paternal homozygous genotype loci on each chromosome, and some heterozygous offspring contained homozygous paternal genotype loci and parental hybrid genotype loci (**Figures 5J–M**). The locus genotype loci of the parent hybrid polyploid heterozygous offspring were biased towards the parent hybrid genotype loci, which was consistent with the flow cytometry results and gene penetration rate analysis. The penetration of inducer line’s genes may have obvious interaction effects with maternal genotypes, and a specific genomic chromosome segment in the offspring was prone to penetration or hybridization.

## 4 Discussion

### 4.1 Interaction effect of the maternal materials and the doubled inducer line during the induction process

Through the correlation analysis of embryo mortality, *cp4-EPSPS* gene loss and SNP typing analysis after pollination with Y3380 for homozygous *B. juncea* 3496, homozygous *B. napus* L. sterile line 0068A, and heterozygous *B. napus* L. 4120, we found that there might be obvious interaction effects between the exogenous gene *cp4-EPSP*S elimination during embryonic development and the maternal genotype Additionally, penetration or hybridization of doubled haploid inducer line gene fragments might also have significant interaction effects with maternal genotypes (Zhang et al., 2021). The homozygous strains in the mother-induced F_1_ were homozygous *B. juncea* 3496 > *B. napus* L. sterile line 0068A > heterozygous *B. napus* L. 4120, which also confirmed that the gene fragment of the doubled haploid inducer line was very different from the corresponding loci gene fragment of the females. The gene fragment of the inducer line was easy to eliminate; when the difference between the gene fragment of the inducer line and that of the females was small or similar, the gene fragment of the inducer line was easy to penetrated or hybridized.

The exogenous gene *cp4-EPSPS* in the inducer line is derived from microorganisms, and there is no homologous gene in common rapeseed species; therefore *cp4-EPSPS* is easily eliminated as a marker gene during the induction process. FISH also showed that the induction process was mainly caused by eliminating paternal chromosomes. When the females were heterozygous, there was an increased possibility of genetic similarity between them and the inducer line or their interaction probability, which increased the likelihood of the paternal gene fragment penetrating or hybridizing. This was observed in the induced offspring of heterozygous 4120, which were hybrid. However, the genetic distance of *B. juncea* 3496 was far from that of Y3380, reaching 0.6, and 3496 was a homozygous material, resulting in a high induction efficiency, low penetration rate, and heterozygosity rate (0.32%–2.80%). In some cases, certain chromosomal segments (A05) were easy to penetrated during the induction process of 3496, indicating that the inducer line and the females had high homology in this segment and were difficult to eliminate. This was consistent with the induction results of paternal chromosome specific elimination during haploid induction in maize and barley (Ravi and Chan, 2010).

### 4.2 Analysis of the mechanism of doubled haploid induction

Studies on the induction mechanism of the inducer line have shown that the selective elimination of paternal chromosomes is the primary cause of induction. According to Li et al. (2017), continuous chromosomal breaks after gametophyte meiosis might may lead to the formation of haploid embryos, as evidenced by mononuclear sequencing. Zhao (Zhao et al., 2013) pollinated the maternal strains using two inducer lines containing the cytogenetic marker B chromosome and an abnormal yellow centromeres H3 fluorescent protein carrier, demonstrating selective chromosome elimination during maize haploid formation. These results provide insights for the studying of the induction mechanism of rapeseed DH induction.

This study used pollen from Y3380 containing the marker gene cp4-EPSPS to pollinate several different types of maternal rapeseed materials. Many embryonic deaths occurred in the dissected rapeseed siliques during embryonic development, and double fertilization was observed 48 h after pollination. We detected the presence and expression of the *cp4-EPSPS* gene in the offspring, and the *cp4* gene was detected on the eighth day after pollination. At this point, the inducer line chromosomes had entered the maternal egg cells and integrate to develop into an embryo. However, with embryonic development, the paternal chromosome continued to be eliminated, leading to a decline in the probability of containing the *cp4-EPSPS* gene, which reached to 0% in the last surviving seeds. The immunofluorescence signal of *cp4* was observed in the seeds on the 13–29th day after pollination, and the proportion of embryos containing fluorescent signals continued to decrease during development. This indicated that the paternal chromosomes were eliminated, and the specific expression of marker genes continued during elimination. The disappearance of gene expression signal (29 d after pollination) occurred earlier than the time of gene elimination (33 d after pollination), indicating that physiological effects during embryonic development caused the elimination of paternal chromosomes and that physiological effects might be caused by inducer line of gene expression (Luo et al., 2021).

The FISH results showed that the induced F_1_ of *B. juncea* (AABB) did not contain the C genome of the inducer line, indicating that the genome of the inducer line was selectively eliminated during the haploid induction process. This is similar to the haploid inducer line in maize, where the inducer line chromosomes are mostly eliminated from the primordium cells within the first week after pollination, leading to haploid formation (Zhao et al., 2013). However, in rapeseed-induced F_1_, 96.52% were identified as tetraploid, whereas the remaining were polyploidy hybridized by parents, and haploids were rarely produced (Fu et al., 2018). Using SNP chip analysis of the induced F_1_, we found that the tetraploid-induced offspring had a penetration of induced line genes, with heterozygosity and homozygosity at the penetration loci. This indicated that the induced line chromosomes were eliminated during embryonic development, and the maternal haploid chromosome or synchronous doubled in the embryo. Because the maternal genotypes were different, the penetration rates of the paternal genes in the offspring were also different.

Analysis of the correlation between the homozygous rate of different females and the probability of induced F_1_ containing *cp4* gene, the effective silique rate, and the average effective fruiting number of induced F_1_ seeds showed that the homozygous loci rate of the females was significantly negatively correlated with the elimination of the induced F_1_ marker gene (r = −0.9997**), whereas the number of fruiting (fruiting rate) of induced F_1_ seeds was significantly positively correlated (r = 0.9999*). These findings indicated that the doubled haploid induction of genomyogenic selectivity was related to the interaction effect of maternal genotypes.

In summary, in the process of rapeseed doubled haploid induction, the maternal egg and the paternal sperm double fertilization formed a zygote, and selective elimination of the induction line genome occurs during zygotic development. The degree of elimination thoroughness was related to the maternal genotype interaction effect. After the paternal genome was eliminated, the zygotic haploid chromosome was doubled synchronously, forming a maternal diploid or parental partial hybrid diploid, such as doubling that occurred before incomplete elimination, and then some paternal gene hybrids would be formed. In the gene editing induced by Y3380 in Li’s study (Li et al., 2021), the edited female was a doubled haploid and did not contain transgenic components; however, the editing target gene loci were heterozygous, indicating that the editing process might occur after the induction of lineage chromosome elimination and maternal chromosome doubling. The results of this study directly demonstrated the possibility of a rapeseed doubled haploid inducer line for gene editing in the maternal genes, editing vector loss, and the acquisition of homozygous individuals of *B. napus* and other Brassicaceae Burnett vegetables.

## 5 Conclusion

In summary, we speculated that the induction process of rapeseed doubled haploids might be due to the inducer line’s chromosome entering the maternal egg cell and forming a zygote through fertilization. During zygotic mitosis, the paternal chromosomes are selectively eliminated, and during the elimination, there might be a phenomenon of partial paternal genes that may penetrate into the maternal genome through homologous exchange. The zygote haploid genome doubled (early haploid doubling, EH phenomenon), and the doubled zygote continued to develop into a complete embryo, finally forming a doubled haploid offspring. The induction rate of the doubled haploid was significantly affected by the maternal genotype and the paternal single plant inducer line, whereas the mortality rate, effective silique rate, and average effective fruiting number of F_1_ seeds were mainly influenced by the maternal genotype. The influencing factors of rapeseed doubled haploid induction and haploid induction in maize are relatively similar, with the difference being that the induced F_1_ in rapeseed is a doubled haploid, whereas it is a haploid in maize. However, this experiment did not exclude the possibility that a few single plants in the offspring of rapeseed doubled haploids were parthenogenetic. This requires further research and demonstration.

## Data availability statement

The original contributions presented in the study are included in the article/**Supplementary Material**. Further inquiries can be directed to the corresponding authors.

## Author contributions

SZ: Investigation, Writing – original draft. LH: Investigation, Data curation, Visualization, Writing – review & editing. QZh: Data curation, Investigation, Visualization, Writing – review & editing. YZ: Investigation, Visualization, Writing – review & editing. MY: Investigation, Writing – review & editing, Formal Analysis, Software. HS: Formal Analysis, Software, Writing – review & editing, Data curation. YL: Data curation, Writing – review & editing, Investigation. JY: Writing – review & editing, Project administration. CL: Writing – review & editing. XG: Writing – review & editing, Investigation. WG: Investigation, Writing – review & editing. JW: Investigation, Writing – review & editing. QZo: Investigation, Writing – review & editing. LT: Investigation, Writing – review & editing. ZK: Investigation, Writing – review & editing. ZL: Writing – review & editing, Resources. CX: Resources, Writing – review & editing. QH: Resources, Writing – review & editing, Funding acquisition. SF: Funding acquisition, Resources, Writing – review & editing, Project administration, Supervision.
