# Supplementary material for: Paternal chromosome elimination of inducer triggers induction of double haploids in Brassica napus
Source: Front Plant Sci. 2023 Oct 30;14:1256338. doi: 10.3389/fpls.2023.1256338 (PMC10642322; doi:10.3389/fpls.2023.1256338)
Supplement: Supplementary file 1 [file DataSheet_1.docx]

**Supplementary Figures**


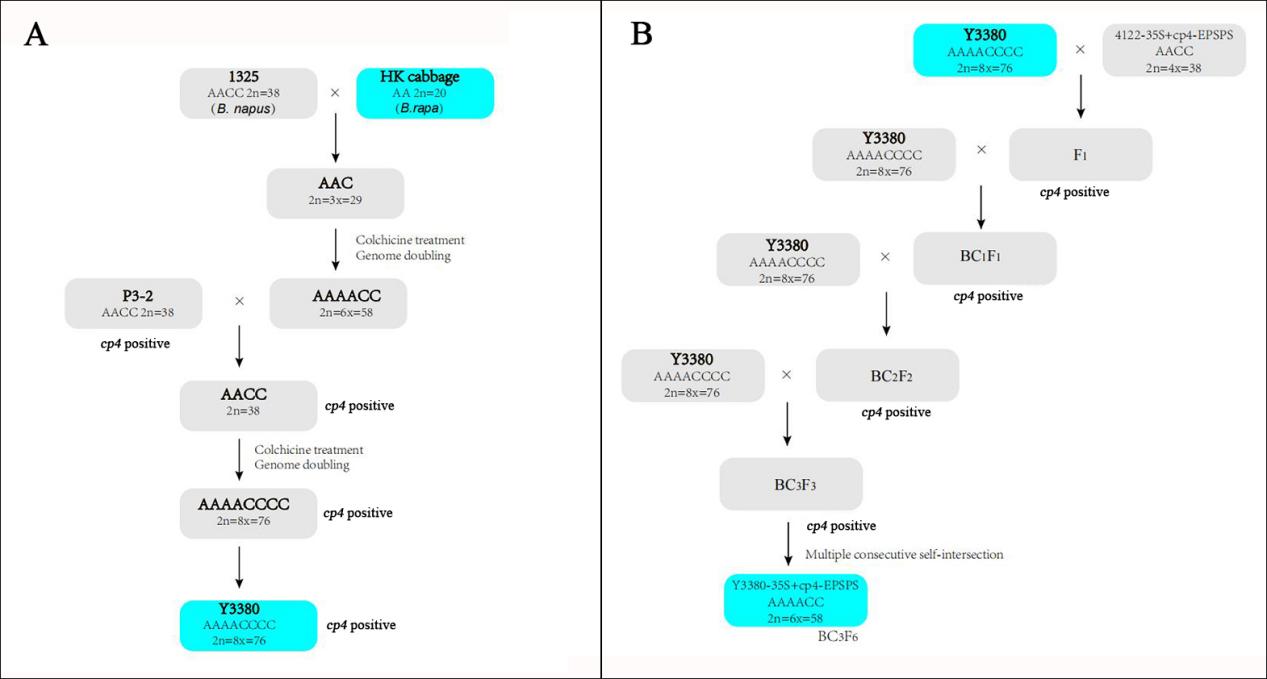


**Fig. S1.** Doubled haploid induction line Y3380 synthesis pathway. (A) Y3380. (B) Y3380-*cp4*.

**
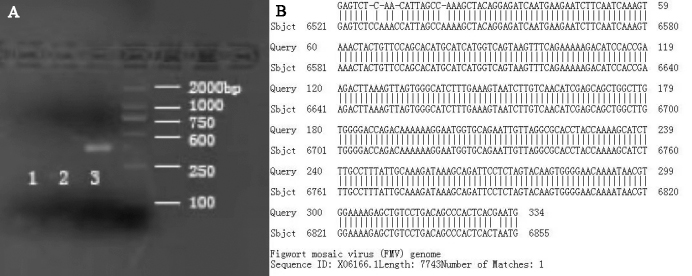
**

**Fig. S2.** *FMV 35S* promoter direct PCR electrophoresis and results compared to known sequences. (A) *FMV 35S* promoter direct PCR electrophoresis diagram. 1 represents blank control *FMV 35S* promoter direct expansion PCR electrophoresis. 2 represent negative control seed *FMV 35S* promoter direct expansion PCR electrophoresis. 3 represents positive control seed *FMV 35S* promoter direct PCR electrophoresis. (B) PCR production compared to known sequences.


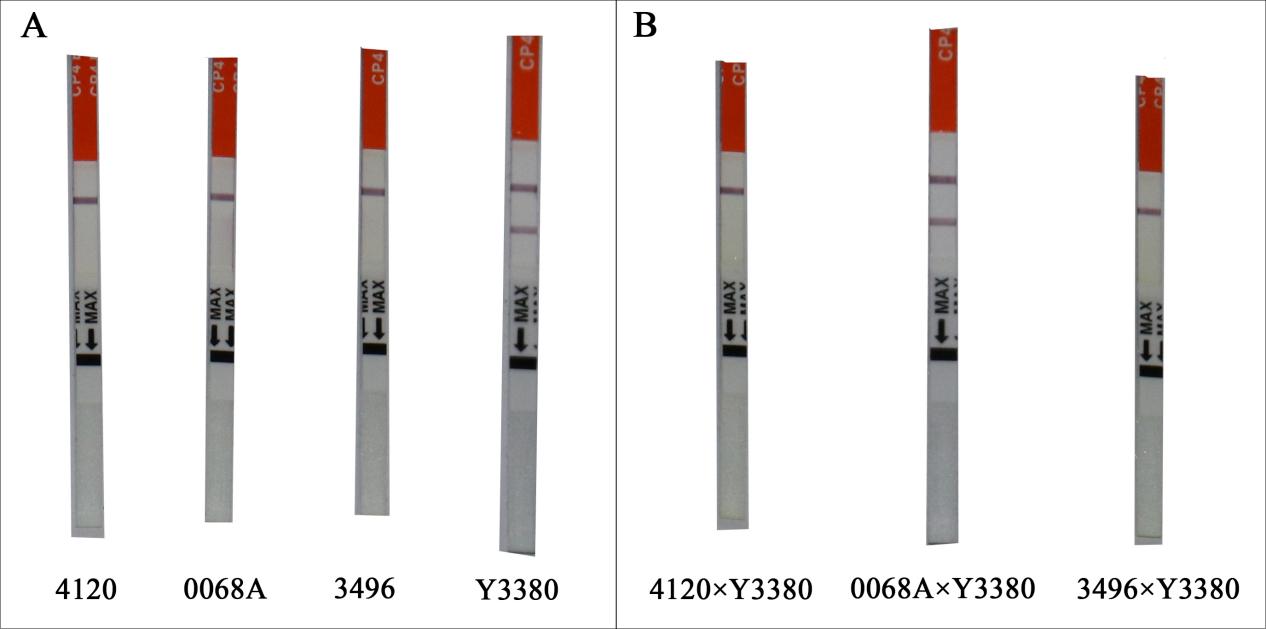


**Fig. S3.** *cp4-EPSPS* gene strip detection of parents and induced F_1_. (A) *cp4-EPSPS* gene strip detection results of parents. (B) *cp4-EPSPS* gene strip detection results of induced F_1_ at 8 d after pollination.

**Supplymentary Tables**

**Table S1**

Number of chromosomes and genotype for parental materials.

| Materials | Genotype | Number of chromosomes |
| --- | --- | --- |
| 3496 | AABB | 2n=4x=36 |
| 4120 | AACC | 2n=4x=38 |
| 0068A | AACC | 2n=4x=38 |
| ZS11 | AACC | 2n=4x=38 |
| 4122 | AACC | 2n=4x=38 |
| 3737 | AACC | 2n=4x=38 |
| Y3380-*cp4* | AAAACC | 2n=6x=58 |

**Table S2**

Primer sequence of *cp4-EPSPS* gene *FMV 35S* promoter.

| Gene | Primer sequence |
| --- | --- |
| *FMV 35S-F* | AGTCCAAAGCCTCAACAAGGTC |
| *FMV 35S-R* | CATTCGTGAGTGGGCTGTCAGG |
| *BnA-F* | CGCCGCTTAACCCTAAGGCTAACAG |
| *BnA-R* | TTCTCTTTAATGTCACGGACGATTT |

**Table S3**

Number of induced F_1_ detected in marker gene tracking and seed mortality rate and survival rate.

| Combination | Days after pollination | N_LS_ | N_LS-_*_cp4_* | N_S_ | N_S-_*_cp4_* | P_LS-_*_cp4_* | P_S-_*_cp4_* | R_SM_ | R_SS_ |
| --- | --- | --- | --- | --- | --- | --- | --- | --- | --- |
| 4120 × Y3380 | 13d | 152 | 132 | 184 | 155 | 86.84% | 84.24% | 24.16% | 75.48% |
|  | 17d | 129 | 123 | 150 | 135 | 95.35% | 90.00% | 40.00% | 60.00% |
|  | 23d | 93 | 90 | 96 | 90 | 96.77% | 93.75% | 62.44% | 37.56% |
|  | 25d | 96 | 77 | 113 | 81 | 80.21% | 71.68% | 59.68% | 40.32% |
|  | 27d | 28 | 24 | 97 | 65 | 85.71% | 67.01% | 90.90% | 9.10% |
|  | 33d | 10 | 0 | 131 | 14 | 0.00% | 10.69% | 96.47% | 3.53% |
| 4120 × ZS11 |  |  |  |  |  |  |  | 5.56% | 94.44% |
| 0068A × Y3380 | 12d | 89 | 78 | 89 | 78 | 87.64% | 87.64% | 36.66% | 63.34% |
|  | 16d | 138 | 111 | 138 | 111 | 80.43% | 80.43% | 48.36% | 51.64% |
|  | 22d | 115 | 52 | 118 | 53 | 45.22% | 44.92% | 41.60% | 58.40% |
|  | 24d | 84 | 4 | 154 | 10 | 4.76% | 6.49% | 59.69% | 40.31% |
|  | 26d | 33 | 0 | 76 | 1 | 0.00% | 1.32% | 87.50% | 12.50% |
|  | 30d | 14 | 0 | 113 | 5 | 0.00% | 4.42% | 96.10% | 3.90% |
| 0068A × ZS11 |  |  |  |  |  |  |  | 5.56% | 93.10% |
| 3496 × Y3380 | 14d | 9 | 8 | 9 | 8 | 88.89% | 88.89% | 43.75% | 56.25% |
|  | 18d | 17 | 13 | 17 | 13 | 76.47% | 76.47% | 45.16% | 54.84% |
|  | 24d | 21 | 15 | 21 | 15 | 71.43% | 71.43% | 38.24% | 61.76% |
|  | 26d | 14 | 5 | 23 | 7 | 35.71% | 30.43% | 53.33% | 46.67% |
|  | 30d | 10 | 0 | 14 | 1 | 0.00% | 7.14% | 72.22% | 27.78% |
|  | 34d | 18 | 0 | 24 | 0 | 0.00% | 0.00% | 66.67% | 33.33% |
|  | 38d | 2 | 0 | 12 | 2 | 0.00% | 16.67% | 95.56% | 4.44% |
| 3496 × 4122 |  |  |  |  |  |  |  | 5.56% | 94.44% |

N_LS_ represents the number of live seeds. N_LS-_*_cp4_* represents the number of live seeds containing *cp4-EPSPS*. N_S_ represents the number of seeds. N_S-_*_cp4_* represents the number of seeds containing *cp4-EPSPS*. P_LS-_*_cp4_* represents the probability diagrams of induced F_1_ live seeds containing *cp4-EPSPS*. P_S-_*_cp4_* represents the probability diagrams of induced F_1_ seeds containing *cp4-EPSPS*. R_SM_ represents seed mortality rate. R_SS_ represents seed survival rate.

**Table S4**

Day temperature after pollination.

| Date | Temperature(℃) | Date | Temperature(℃) | Date | Temperature(℃) |
| --- | --- | --- | --- | --- | --- |
| 12/03/2019 | 20-11 | 26/03/2019 | 22-12 | 09/04/2019 | 28-18 |
| 13/03/2019 | 28-9 | 27/03/2019 | 23-15 | 10/04/2019 | 19-14 |
| 14/03/2019 | 17-11 | 28/03/2019 | 28-16 | 11/04/2019 | 21-14 |
| 15/03/2019 | 20-8 | 29/03/2019 | 27-17 | 12/04/2019 | 18-11 |
| 16/03/2019 | 17-10 | 30/03/2019 | 22-15 | 13/04/2019 | 20-15 |
| 17/03/2019 | 18-8 | 31/03/2019 | 22-13 | 14/04/2019 | 23-14 |
| 18/03/2019 | 20-11 | 01/04/2019 | 21-11 | 15/04/2019 | 20-15 |
| 19/03/2019 | 20-12 | 02/04/2019 | 21-13 | 16/04/2019 | 27-17 |
| 20/03/2019 | 24-16 | 03/04/2019 | 22-11 | 17/04/2019 | 28-17 |
| 21/03/2019 | 19-11 | 04/04/2019 | 20-14 | 18/04/2019 | 30-20 |
| 22/03/2019 | 17-11 | 05/04/2019 | 27-16 | 19/04/2019 | 22-20 |
| 23/03/2019 | 18-8 | 06/04/2019 | 31-19 | 20/04/2019 | 20-10 |
| 24/03/2019 | 17-7 | 07/04/2019 | 30-20 | 21/04/2019 | 28-18 |
| 25/03/2019 | 19-10 | 08/04/2019 | 30-19 | 22/04/2019 | 28-20 |

**Table S5**

Flow cytometric identification of induced F_1_.

| Sample | Number | G1 peak (D thousand line) | Average G1 peak ( D thousand line) | Haploid number | Tetraploid number | Polyploid number |
| --- | --- | --- | --- | --- | --- | --- |
| Y3380 | 4 | 625.8-689.9 | 649.2 | 0 | 0 | 4 |
| 4120 | 5 | 402.4-424.7 | 410.5 | 0 | 5 | 0 |
| 0068A | 5 | 340.6-357.0 | 346.6 | 0 | 5 | 0 |
| 3496 | 5 | 413.8-469.4 | 444.7 | 0 | 5 | 0 |
| 4120 × Y3380 | 6 | 416.9-523.9 | 451.7 | 0 | 4 | 2 |
| 0068A × Y3380 | 87 | 354.0-623.8 | 404.2 | 0 | 85 | 2 |
| 3496 × Y3380 | 21 | 305.5-378.7 | 348.3 | 0 | 21 | 0 |

**Table S6**

Sample for SNP detection.

| Sample | Origin |
| --- | --- |
| Z20-1, Z20-2, Z20-3 | 0068A×Y3380-44-7 |
| Y3380-44-7 | Hexaploid (AAAACC) |
| Z21-1, Z21-2, Z21-3 | 0068A×Y3380-44-19 |
| Y3380-44-19 | Hexaploid (AAAACC) |
| Z22-1, Z22-2 | 0068A×ZS11 |
| ZS11 | Tetraploid *Brassica Napus* L. (AACC) |
| 0068A-1, 0068A-2 | *pol CMS Brassica Napus* L.（(AACC) |
| Z23-1, Z23-2, Z23-3, Z23-4, Z23-5 | 3496×Y3380-50-21 |
| Y3380-50-21 | Hexaploid (AAAACC) |
| Z24-1, Z24-2 | 3496×4122 |
| 4122 | Hexaploid *Brassica Napus* L. (AACC) |
| 3496-1, 3496-2 | Hexaploid *Brassica juncea* (AABB) |
| Z25-1, Z25-2, Z26-1, Z27-1 | 4120×Y3380-44-7 |
| Z28-1 | 4120×Y3380-44-19 |
| Z29-1, Z29-2 | 4120×ZS11 |
| 4120-1 (F_1_), 4120-2 (F_1_) | ZS11×6170 |
| 6170 | Tetraploid *Brassica Napus* L. (AACC) |
| 5000 | *Brassica Napus* L. ZS11 |
| 4000 | *Brassica Napus* L. 4122 |

**Table S7**

Rate of parental gene penetration in offspring.

| Offspring sample | Paternal gene penetration rate (%) | Maternal gene penetration rate (%) | Homozygous loci rate (%) |
| --- | --- | --- | --- |
| Z20-1 | 0 | 99.56 | 96.61 |
| Z20-2 | 0 | 99.63 | 96.64 |
| Z20-3 | 0 | 99.42 | 96.71 |
| Z21-1 | 3.99 | 71.74 | 84.36 |
| Z21-2 | 2.78 | 83.12 | 87.20 |
| Z21-3 | 7.84 | 69.75 | 85.47 |
| Z22-1 | 5.31 | 4.56 | 67.86 |
| Z22-2 | 5.18 | 4.75 | 67.79 |
| Z23-1 | 0.32 | 97.33 | 96.45 |
| Z23-2 | 0.69 | 96.87 | 97.08 |
| Z23-3 | 0.76 | 97.14 | 97.09 |
| Z23-4 | 2.86 | 88.34 | 90.58 |
| Z23-5 | 0.79 | 96.13 | 95.64 |
| Z25-1 | 9.92 | / | 84.06 |
| Z25-2 | 12.43 | / | 86.23 |
| Z26-1 | 35.42 | / | 95.89 |
| Z27-1 | 29.88 | / | 61.09 |
| Z28-1 | 35.49 | / | 63.98 |

**Table S8**

SNP genotyping statistics.

| Sample type | Sample Number | G1 peak (D thousand line) | Heterozygous loci rate (%) | Homozygous loci rate (%) |
| --- | --- | --- | --- | --- |
| 0068A | 0068A-1 | 345.9 | 3.33 | 96.67 |
| 0068A × Y3380 | Z20-1 | 387.5 | 3.39 | 96.61 |
|  | Z20-2 | 374.0 | 3.36 | 96.64 |
|  | Z20-3 | 448.0 | 3.29 | 96.71 |
|  | Z21-1 | 418.4 | 15.64 | 84.37 |
|  | Z21-2 | 387.4 | 12.80 | 87.20 |
|  | Z21-3 | 402.9 | 14.53 | 85.48 |
| ZS11 | ZS11 | 426.2 | 2.51 | 97.49 |
| 0068A × ZS11 | Z22-1 | 406.8 | 32.14 | 67.86 |
|  | Z22-2 | 405.7 | 32.21 | 67.79 |
| 3496 | 3496-1 | 440.7 | 2.81 | 97.19 |
|  | 3496-2 | 436.8 | 3.83 | 96.17 |
| 3496 × Y3380 | Z23-1 | 372.2 | 3.55 | 96.45 |
|  | Z23-2 | 360.5 | 2.92 | 97.08 |
|  | Z23-3 | 374.7 | 2.91 | 97.09 |
|  | Z23-4 | 342.3 | 9.42 | 90.58 |
|  | Z23-5 | 354.1 | 4.36 | 95.64 |
| 4122 | 4122 | 403.5 | 2.54 | 97.46 |
| 3496 × 4122 | Z24-1 | 372.8 | 18.56 | 81.45 |
|  | Z24-2 | 353.2 | 18.61 | 81.39 |
| 4120 | 4120-1 | 418.0 | 22.86 | 77.14 |
|  | 4120-2 | 402.4 | 26.71 | 73.29 |
| 4120 × Y3380 | Z25-1 | 416.9 | 15.94 | 84.06 |
|  | Z25-2 | 421.7 | 13.77 | 86.23 |
|  | Z26-1 | 429.9 | 4.11 | 95.89 |
|  | Z27-1 | 523.9 | 38.91 | 61.09 |
|  | Z28-1 | 495.9 | 36.02 | 63.98 |
| 4120 × ZS11 | Z29-1 | 410.1 | 17.74 | 82.26 |
|  | Z29-2 | 406.2 | 17.43 | 82.57 |
| Y3380 | Y3380-44-7 | 671.0 | 10.94 | 89.06 |
|  | Y3380-44-19 | / | 14.67 | 85.33 |
|  | Y3380-50-21 | 787.9 | 13.69 | 86.31 |
